# Differential Regulation of Gene Expression in Lung Cancer Cells by Diacyglycerol-Lactones and a Phorbol Ester Via Selective Activation of Protein Kinase C Isozymes

**DOI:** 10.1038/s41598-019-42581-4

**Published:** 2019-04-15

**Authors:** Mariana Cooke, Victoria Casado-Medrano, Jihyae Ann, Jeewoo Lee, Peter M. Blumberg, Martin C. Abba, Marcelo G. Kazanietz

**Affiliations:** 1 Department of Systems Pharmacology and Translational Therapeutics, Perelman School of Medicine, University of Pennsylvania, Philadelphia, PA 19104 USA; 2 Laboratory of Medicinal Chemistry, College of Pharmacy, Seoul National University, Seoul, 08826 Republic of Korea; 3 Laboratory of Cancer Biology and Genetics, Center for Cancer Research, NCI, Bethesda, MD 20892 USA; 4 Centro de Investigaciones Inmunológicas Básicas y Aplicadas, Universidad Nacional de La Plata, CP1900 La Plata, Argentina

## Abstract

Despite our extensive knowledge on the biology of protein kinase C (PKC) and its involvement in disease, limited success has been attained in the generation of PKC isozyme-specific modulators acting via the C1 domain, the binding site for the lipid second messenger diacylglycerol (DAG) and the phorbol ester tumor promoters. Synthetic efforts had recently led to the identification of AJH-836, a DAG-lactone with preferential affinity for novel isozymes (nPKCs) relative to classical PKCs (cPKCs). Here, we compared the ability of AJH-836 and a prototypical phorbol ester (phorbol 12-myristate 13-acetate, PMA) to induce changes in gene expression in a lung cancer model. Gene profiling analysis using RNA-Seq revealed that PMA caused major changes in gene expression, whereas AJH-836 only induced a small subset of genes, thus providing a strong indication for a major involvement of cPKCs in their control of gene expression. *MMP1*, *MMP9*, and *MMP10* were among the genes most prominently induced by PMA, an effect impaired by RNAi silencing of PKCα, but not PKCδ or PKCε. Comprehensive gene signature analysis and bioinformatics efforts, including functional enrichment and transcription factor binding site analyses of dysregulated genes, identified major differences in pathway activation and transcriptional networks between PMA and DAG-lactones. In addition to providing solid evidence for the differential involvement of individual PKC isozymes in the control of gene expression, our studies emphasize the importance of generating targeted C1 domain ligands capable of differentially regulating PKC isozyme-specific function in cellular models.

## Introduction

Protein kinase C (PKC) isozymes represent a family of related serine-threonine kinases that have been broadly implicated in cancer progression. These kinases are key constituents of signal transduction pathways that control cellular functions such as proliferation, survival, differentiation, motility, and gene expression. PKCs are classified into three groups based on their biochemical and structural properties: “classical/conventional” (calcium-dependent cPKCs α, βI, βII and γ), “novel” (calcium-independent nPKCs δ, ε, η and θ), and “atypical” (calcium-independent aPKCs ζ and ι). cPKCs and nPKCs are the best characterized targets for diacylglycerol (DAG), a lipid second messenger generated by stimulation of membrane receptors, whereas aPKCs are unresponsive to DAG^1–3^.

Consistent with the contrasting effects of individual PKC isozymes in promoting or suppressing tumor growth, it is now widely recognized that discrete members of this family can have either similar or opposite roles in a cellular context^4–6^. For example, early studies in fibroblasts showed inhibitory and promoting roles for PKCδ and PKCε, respectively, in cell growth and tumorigenesis^7^. Notwithstanding, effects mediated by individual PKC isozymes are strictly dependent on the cell type and context. An extra level of complexity in PKC biology involves the causal relationship between isozyme expression and disease progression. Most notably, PKCε has been largely described as an oncogenic kinase and a cancer progression biomarker. Indeed, PKCε is overexpressed in numerous cancer types, including lung, prostate and breast cancer^8–12^, and silencing its expression causes major disruption in the ability of cancer cells to grow and invade^10,13,14^. Experiments using transgenic and knock-out mouse models greatly support the involvement of PKCε in tumor development, growth, and metastasis^13,15^. Specifically in human non-small cell lung cancer (NSCLC) cells, PKCε is associated with mitogenicity, tumorigenic potential, and the ability of the cells to migrate, invade and metastasize^16,17^.

Binding of DAG occurs at the C1 domains^18^, motifs expressed in tandem in the regulatory regions of both cPKCs and nPKCs. This binding represents the key step for allosteric activation of the enzyme and phosphorylation of intracellular substrates^2,19,20^. Natural products such as phorbol esters also bind to PKC C1 domains with high affinity, as determined many years ago by means of radiolabeled phorbol ester competition assays^21,22^. Structural studies established that DAGs and phorbol esters cap a hydrophilic groove between two pulled-apart beta-strands at the tip of the C1 domain, thus creating a contiguous hydrophobic surface that allows the protein to insert into the plasma membrane. This association is further stabilized by additional interactions between charged residues on the surface of the C1 domain and the headgroups of membrane acidic phospholipids^23–25^. In addition to PKCs, a number of other proteins with C1 domains are capable of binding DAG and phorbol esters with high affinity, including protein kinase D (PKD1, 2, and 3), lipid kinases (DAG-kinases/DGKs), regulators of small GTPases (both RasGRPs and chimaerins - the former a RasGEF, the latter a Rac-GAP), and proteins involved in exocytosis/trafficking (Munc-13 isozymes)^1,3^.

A major roadblock in the development of isozyme-selective PKC ligands is the remarkable similarity among C1 domains. Phorbol esters and related compounds, such as bryostatins and deoxyphorbol esters, have similar affinities *in vitro* for individual PKC isozymes^18,22^. However, these agents exert distinctive biological responses in cellular and animal models^26–28^, possibly reflecting their differential ability to insert into membranes, the formation of particular protein-ligand-membrane ternary complexes, and/or specific ligand interactions with lipid microdomains^29–31^. Conceptually, this diversity in ligand response underscores rich opportunities for generating synthetic compounds capable of selectively targeting individual PKCs. To-date, a powerful approach to accomplish this goal has been the cyclization of the flexible glycerol backbone of natural DAGs into rigid lactone structures. The DAG-lactone provides a scaffold that can be easily manipulated to introduce diverse DAG side chains, thereby conferring biological diversity, as demonstrated by means of combinatorial libraries with DAG-lactones with different acyl- and alkyl-substitutions^32–34^.

Recently, we developed DAG-lactones with the incorporation of linoleic acid derivatives as well as saturated and unsaturated alkyl chain substitutions, which provided compounds with preferential affinity for nPKCs^35,36^. One of these C1 domain ligands, the DAG-lactone (E)-(2-(hydroxymethyl)-4-(3-isobutyl-5-methylhexylidene)-5-oxotetrahydrofuran-2-yl) methyl pivalate (AJH-836), has marked selectivity *in vitro* for PKCδ and PKCε relative to PKCα and PKCβ. This pattern differs substantially from that of the prototypical phorbol esters and natural DAG which display no discernible selectivity^36^. Characterization of AJH-836 in a biological setting revealed selective translocation of PKCε to the plasma membrane (a readout of activation) relative to PKCα. Moreover, AJH-836 induced major changes in the reorganization of the actin cytoskeleton in lung cancer cells, a response that is mediated primarily by PKCε as determined by means of isozyme specific RNAi and pharmacological approaches^36^. This unique selectivity pattern of AJH-836 can be pharmacologically exploited for the study of PKC isozyme-specific cellular responses in these cells.

It is well known that phorbol esters induce profound changes in the expression of genes as well as in the activation of signaling pathways that ultimately impact on transcriptional regulation^37,38^. Based on the selectivity of AJH-836, in the present study we wished to test the hypothesis that this DAG-lactone induces a characteristic pattern of changes in gene expression that is distinct from that of a prototypical phorbol ester. Our results revealed major differences in the transcriptional activation and repression of genes in a lung cancer model, providing strong evidence for the differential involvement of PKC isozymes in the control of gene expression in this system, and further emphasizing the importance of generating targeted C1 domain ligands to dissect isozyme-specific biological outcomes.

## Results

### Transcriptome analysis of A549 cells treated with PMA and DAG-lactones

**Figure 1:**
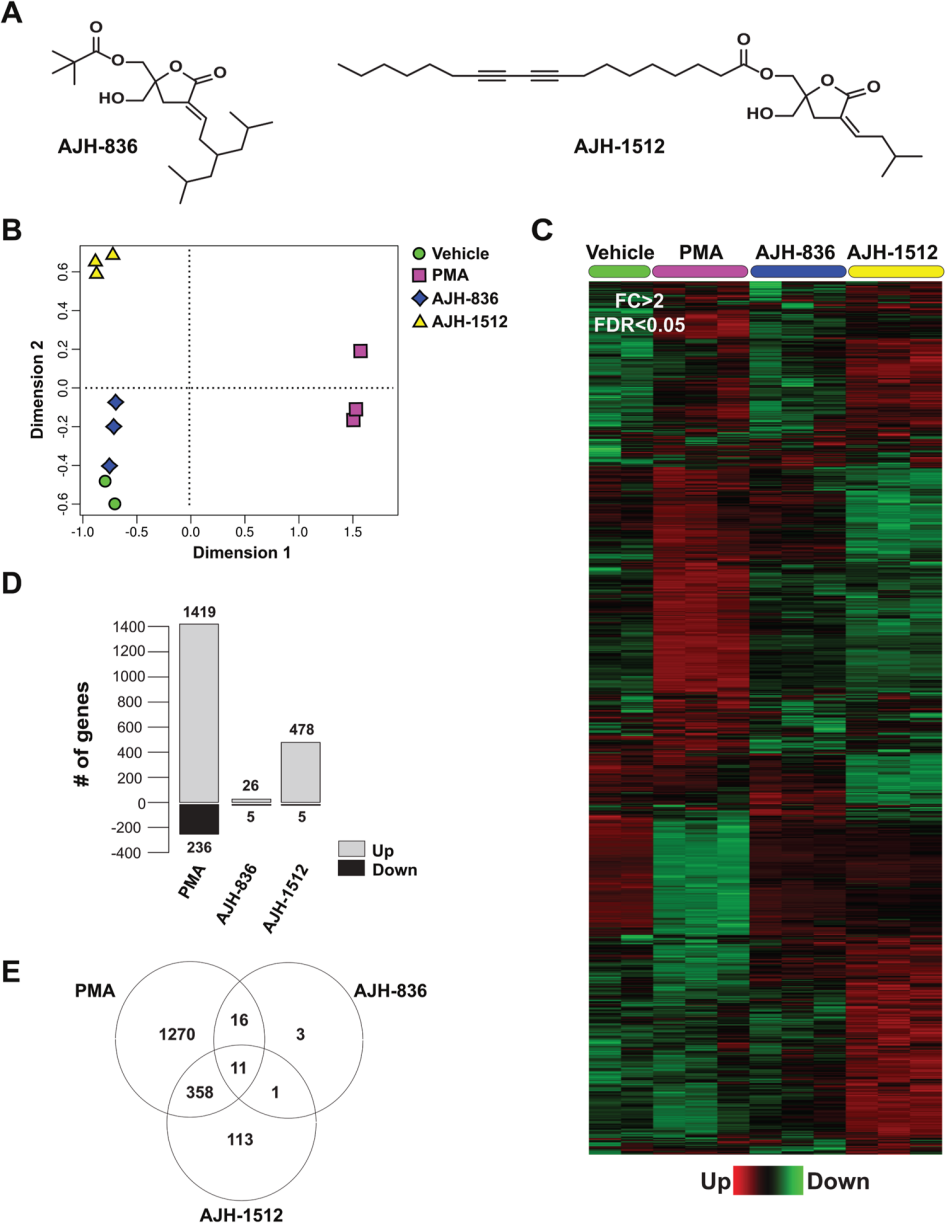
Gene expression profiling of PMA, AJH-836 and AJH-1512 in A549 lung cancer cells. (**A**) Structures of DAG-lactones AJH-836 and AJH-1512. (**B**) Multidimensional scaling plot showing the distance of each sample from each other determined by their RNA-Seq profiles. The leading logFC is the distance metric used in both dimensions and represents the average (root mean square) of the largest absolute logFC between each pair of samples. (**C**) Heatmap of 1772 deregulated genes in A549 cells subjected to the different treatments (Fold-change > 2; FDR < 0.05). The most significant transcript isoform for each gene was employed for heatmap visualization. The color scale at the bottom of the heatmap is used to represent expression level (*green*, low expression; *red*, high expression). (**D**) Number of genes differentially up-regulated and down-regulated among compounds. (**E**) Venn diagram of transcripts commonly modulated among treatments.

A previous study described that incorporation of linoleic acid derivatives as well as saturated and unsaturated alkyl substitutions into the side chains of DAG-lactones confers higher affinity for the novel PKCε relative to the classical PKCα^35^. One of these compounds, the DAG-lactone AJH-836 (Fig. 1A), displays remarkable selectivity for nPKCs relative to cPKCs *in vitro* and in cellular models, unlike the prototypical phorbol ester PMA (phorbol 12-myristate 13-acetate) that equally activates cPKCs and nPKCs^36^. Towards our objective of establishing whether the selectivity of C1 domain ligands could be reflected in differential biological outcomes, we decided to compare the ability of AJH-836 and PMA to modulate gene expression.

A global gene expression profiling was carried out in A549 lung adenocarcinoma cells treated with either AJH-836 or PMA. A549 cells express cPKCα and nPKCs δ and ε, as well as DAG/PMA-unresponsive aPKCs ζ and ι^16,36^ (data not shown). For this analysis, we used a concentration of AJH-836 (1 μM) that fully activates PKCε with only marginal activation of PKCα. As part of the experimental design, we included the related DAG-lactone (*Z*)-(2-(hydroxymethyl)-4-(3-methylbutylidene)-5-oxotetrahydrofuran-2-yl) methyl octadeca-9,11-diynoate (known as AJH-1512) (Fig. 1A). Although AJH-1512 binds equally to cPKCs and nPKCs, at the concentration used in this study (1 μM), it only causes a minimal activation of PKCα and PKCε^36^. Treatment was carried out for 1 h, and RNA for analysis was extracted 3 h later. The reason for using this protocol was two-fold: first, the short-time treatment allows the analysis of early genes directly regulated by these ligands rather than a secondary wave of gene transcription; second, this protocol avoided the down-regulation of PKCs which is normally observed at longer times of exposure to PMA and AJH-836, as we recently described^36^. Comparison of transcriptome profiling was conducted to identify genes differentially regulated by each treatment relative to vehicle using the edgeR test (FDR < 0.05). A 2-fold change relative to vehicle-treated cells was used as a cut-off. Comparison of gene expression upon PMA, AJH-836 and AJH-1512 treatment under the above conditions revealed substantial differences, as shown in the multidimensional plot (Fig. 1B) and the heatmap of dysregulated transcripts (Fig. 1C). As predicted, PMA induced major changes in gene expression in A549 cells. Indeed, 1655 genes displayed statistically significant changes, with 1419 genes up-regulated and 236 genes down-regulated (Fig. 1D and Suppl. Table 1). Remarkably, AJH-836 caused only a very small effect on gene expression. As shown in Fig. 1D, AJH-836 significantly changed the expression of 31 genes (26 up-regulated and 5 down-regulated), of which 87% (27 genes) overlapped with PMA-regulated genes (Fig. 1E). With regard to AJH-1512, this DAG-lactone induced a much larger change in gene expression (483 genes) relative to AJH-836, with 478 genes up-regulated and 5 genes down-regulated (Fig. 1D). Of the genes regulated by AJH-1512, 76% (369 genes) overlapped with PMA-regulated genes and 2% (12 genes) overlapped with AJH-836-regulated genes (Fig. 1D).

**Figure 2:**
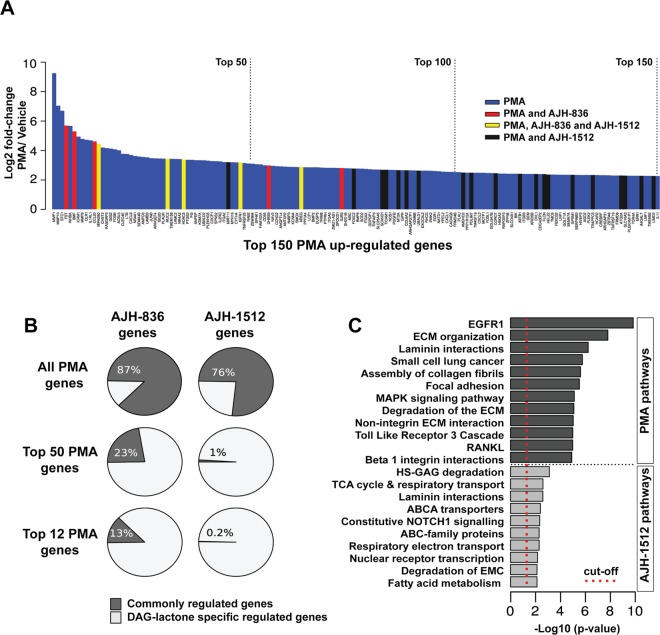
Comparative gene expression signatures among compounds and their modulated signaling pathways. (**A**) Top 150 PMA up-regulated genes and their overlap with the AJH-836 and AJH-1512 up-regulated genes. (**B**) Percentage of genes commonly modulated by AJH-836 (*left pie charts*) and AJH-1512 (*right pie charts*) in comparison to the PMA signature (all PMA-regulated genes), the top 50, and the top 12 PMA regulated genes. (**C**) Functional enrichment analysis of differentially expressed genes among compounds.

Next, we performed a comparative qualitative analysis of the genes regulated by PMA and the DAG-lactones. Figure 2A depicts the top 150 PMA-up-modulated genes, in which those regulated by the DAG-lactones are highlighted. This figure showed considerable differences in the distribution of AJH-836- and AJH-1512-regulated genes within this subset. Notably, several genes regulated by AJH-836 distributed among the top PMA-up-regulated genes, while the AJH-1512-regulated genes are mostly represented in “tail” of the figure. This distinctive distribution can be also appreciated in the pie charts in Fig. 2B, which show that 23% of the AJH-836 up-regulated genes (7/31) are included in the top 50 PMA-up-regulated genes, whereas only 1% of the AJH-1512 up-regulated genes (5/483) are comprised in this group. Moreover, narrowing down this analysis to the top 12 PMA-up-regulated genes revealed that there is an overlap of 13% (4/31) for AJH-836 and only 0.2% for AJH-1512 (1/483). A list of the top 25 genes up-regulated by PMA and the DAG-lactones are listed in Table 1. A noticeable finding is that three metalloproteases widely implicated in the remodeling of the extracellular matrix, invasion and metastasis, are included among the top PMA-up-regulated genes. Specifically, the RNA-Seq analysis revealed a robust up-regulation of *MMP1* (600-fold induction), *MMP9* (27-fold induction) and *MMP10* (131-fold induction) by PMA.

**Table 1:** Genes regulated by PMA, AJH-836, and AJH-1512. The top 25 up-regulated genes for each treatment are shown.

PMA	AJH-836	AJH-1512
MMP1	FST	MIR711
MMP10	GJB3	PSMG4
STC1	BMF	DPM3
FST	SPEG	SPEG
INHBA	MIR4260	SCAND1
BMF	HDAC5	GPSM1
ICAM1	PSMG4	TRAPPC5
MMP9	MUC2	C14ORF80
OLR1	IGFN1	GNB1L
IL1RL1	NIPAL1	CHTF18
CCL20	SYT12	C9ORF16
MIR4260	TINCR	BRICD5
CHST2	PLEKHA4	MCRIP2
RASGRP3	DHRS9	MIR937
GOS2	ROS1	AMDHD2
FOSB	EDN2	C2ORF81
ACSL5	DPM3	AMIGO3
CLEC4E	DOC2A	IFITM3
LTB	SPTB	EVI5L
CXCL8	CCL20	MIF
NR4A1	MIR614	LRFN4
SEMA4B	PDLIM2	NME3
AMPD3	SOX8	TREX1
LAMB3	RELL2	MZT2A
JUNB	AREG	MIB2

Figure 2C shows the functional enrichment analysis of specific bioprocess/signaling pathways for PMA and AJH-1512 gene expression signatures. PMA treatment was associated with the modulation of the EGFR1 (p = 1.49 E-10), MAPK (p = 7.86 E-6), Toll like receptor 3 (p = 1.05 E-5) and RANKL (p = 1.05 E-5) signaling pathways, as well as with extracellular matrix remodeling and cell–matrix adhesion properties. AJH-1512 signature was mainly associated with the modulation of metabolic processes such as glycosaminoglycan degradation (p = 0.0008), tricarboxylic acid cycle and respiratory electron transport (p = 0.002) and fatty acid metabolism (p = 0.007). The low number of genes modulated by AJH-836 precluded from obtaining a statistically significant functional enrichment analysis of the signature for this compound.

**Figure 3:**
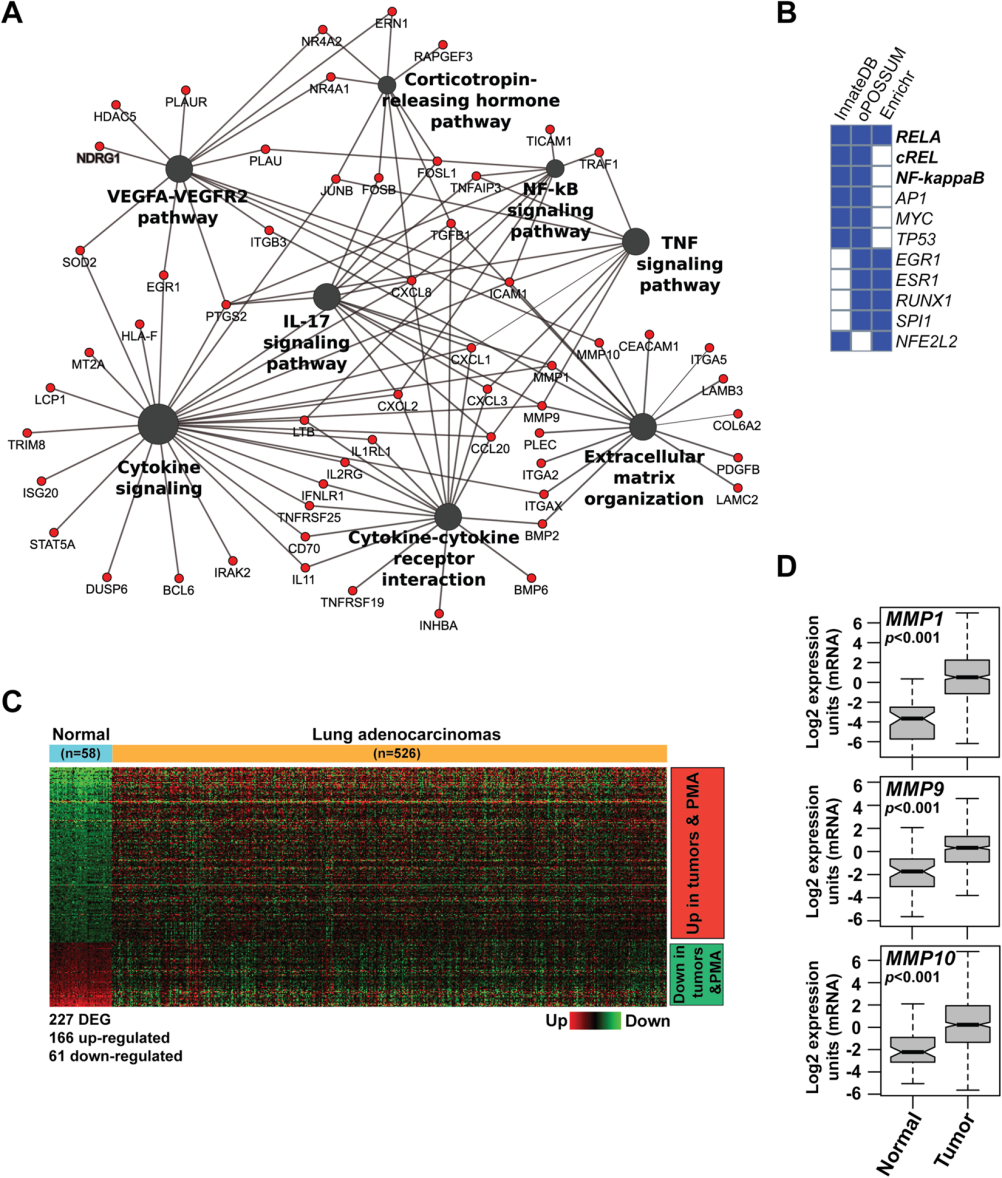
Functional analysis of PMA-regulated genes. (**A**) Network of functionally enriched pathways and genes differentially expressed by PMA treatment relative to vehicle. *Red*, up-regulated genes. (**B**) Comparative enrichment analysis of putative transcription factor binding sites (TFBS) across the promoter of PMA-regulated genes, using three different resources (InnateDB, oPOSSUM and Enrichr). (**C**) Heatmap comparing the PMA up- and down-regulated genes with those in human lung adenocarcinomas, using the TCGA Lung Adenocarcinoma (LUAD) dataset. (**D**) Up-regulation of *MMP1*, *MMP9* and *MMP10* genes in human lung adenocarcinomas relative to normal tissue, according to the TCGA Lung Adenocarcinoma (LUAD) dataset (p < 0.001).

Next, we analyzed the network of functionally enriched pathways and genes differentially expressed in the PMA treatment compared to vehicle. Interestingly, we observed a significant enrichment in pathways related to cytokine/chemokine signaling, effectors of cytokine responses, such as the NF-κB pathway, and components of the extracellular matrix organization (e.g. *MMP1*, *MMP9*, and *MMP10*) (Fig. 3A). A similar analysis for AJH-1512 revealed a significantly different network profile that included pathways involved in focal adhesions, ABC transporters, and Notch signaling (Fig. S1), as expected from the differences observed between PMA and this DAG-lactone in the functional enrichment analysis of bioprocesses and signaling pathways.

To identify transcriptional regulatory networks, we performed a comparative enrichment analysis of putative transcription factor binding sites (TFBS) across the promoters of PMA-regulated genes, using three different resources (InnateDB, oPOSSUM and Enrichr). This analysis, which establishes the over-representation of transcription factors in databases that most likely regulate PMA-associated gene expression, identified statistically significant enrichment of many cancer-related transcription factors. These include NF-κB family members, AP1, MYC and TP53 gene targets (Fig. 3B).

Comparison of PMA-regulated genes in A549 adenocarcinoma cells with those of TCGA lung adenocarcinoma datasets demonstrated significant overlapping of 227 genes (166 up-regulated and 61 down-regulated) (Fig. 3C and Suppl. Table 2). Among these genes, human lung adenocarcinomas showed significant up-regulation of *MMP1* (~24-fold), *MMP9* (~7-fold) and *MMP10* (~4-fold) (Fig. 3D and Suppl. Table 2).

### Analysis of genes differentially regulated by PMA and AJH-836

**Figure 4:**
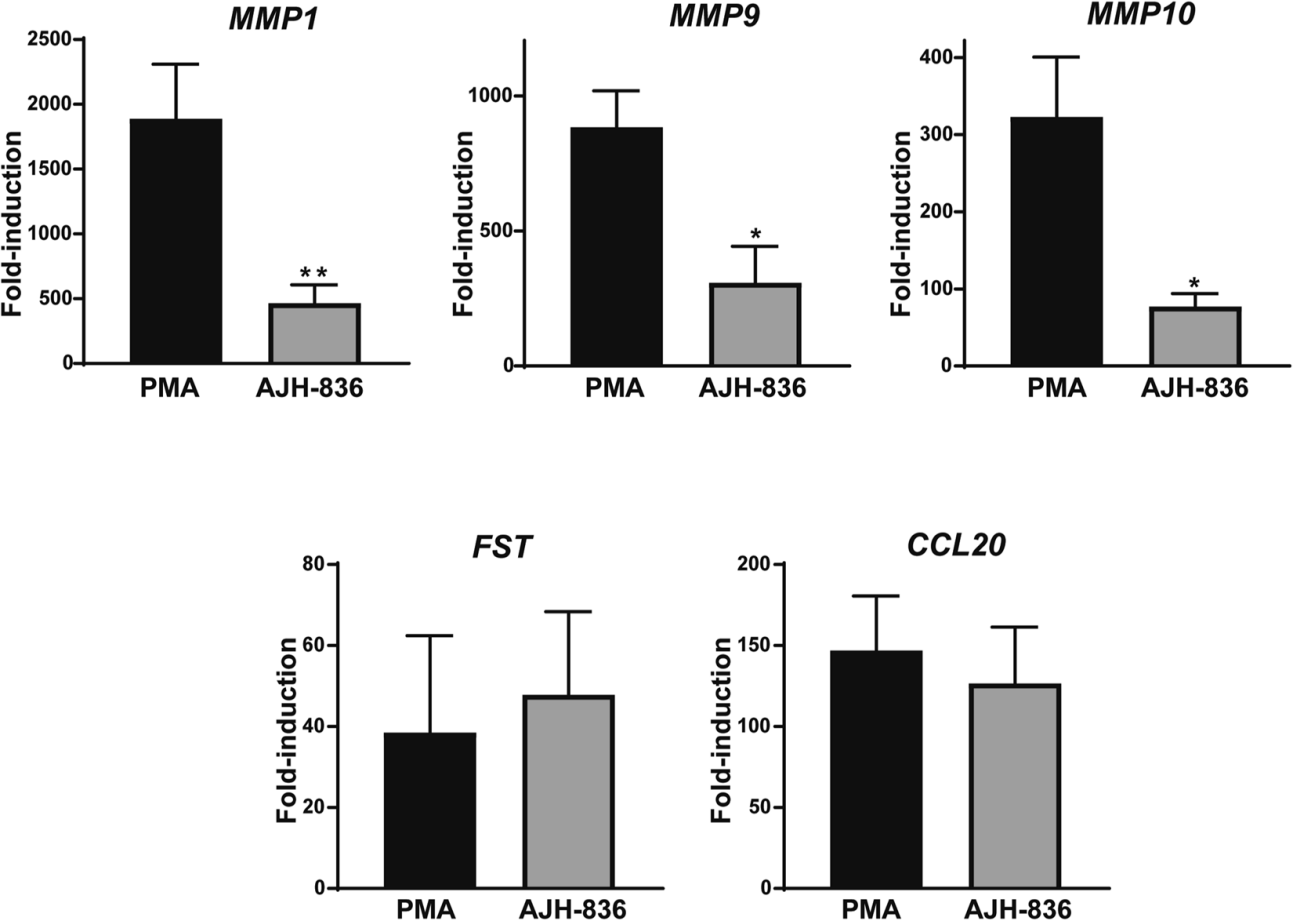
Differential induction of genes by PMA and AJH-836. Serum-starved A549 cells were treated with either PMA (0.1 μM) or AJH-836 (1 μM) for 1 h. RNA was extracted 3 h later, reversed transcribed to cDNA, and used for Q-PCR analysis for the indicated genes. Results are expressed as fold-induction relative to cells treated with vehicle. Data represents the mean ± S.E.M. of 5 independent experiments. ^*^p < 0.05; ^**^p < 0.01 *vs*. PMA.

As the next step, we validated results obtained from the RNASeq analysis using Q-PCR. For this purpose, we selected 5 of the top 12 PMA-up-regulated genes known to be involved in lung cancer progression. Specifically, we analyzed *MMP1*, *MMP9*, and *MM-10*, *FST* (follistatin, a lung cancer biomarker produced by lung cancer cells), and *CCL20* (a chemokine involved in the pathogenesis of lung cancer and other cancers)^39,40^. A549 cells were treated with PMA, AJH-836 or vehicle for 1 h, and 3 h later RNA was extracted and reversed transcribed. cDNA was used for Q-PCR analysis with specific probes. As shown in Fig. 4, PMA caused a large induction in the expression of *MMP1* (~1,800-fold), *MMP9* (~900-fold), and *MMP10* (~300-fold), arguing for a major role for PKCs in metalloprotease induction. In addition, PMA up-regulated *FST* and *CCL20* expression by ~40-fold and ~150-fold, respectively. The induction of *MMP1*, *MMP9*, and *MMP10* by AJH-836, was significantly lower than that caused by PMA. On the other hand, *FST* and *CCL20* were induced to similar extents by AJH-836 and PMA. These results recapitulate those observed in the RNA Seq analysis showing that metalloprotease genes were induced preferentially by PMA, whereas *FST* and *CCL20* were markedly up-regulated both by PMA and AJH-836.

### Differential involvement of PKC isozymes in metalloprotease gene induction in A549 cells

**Figure 5:**
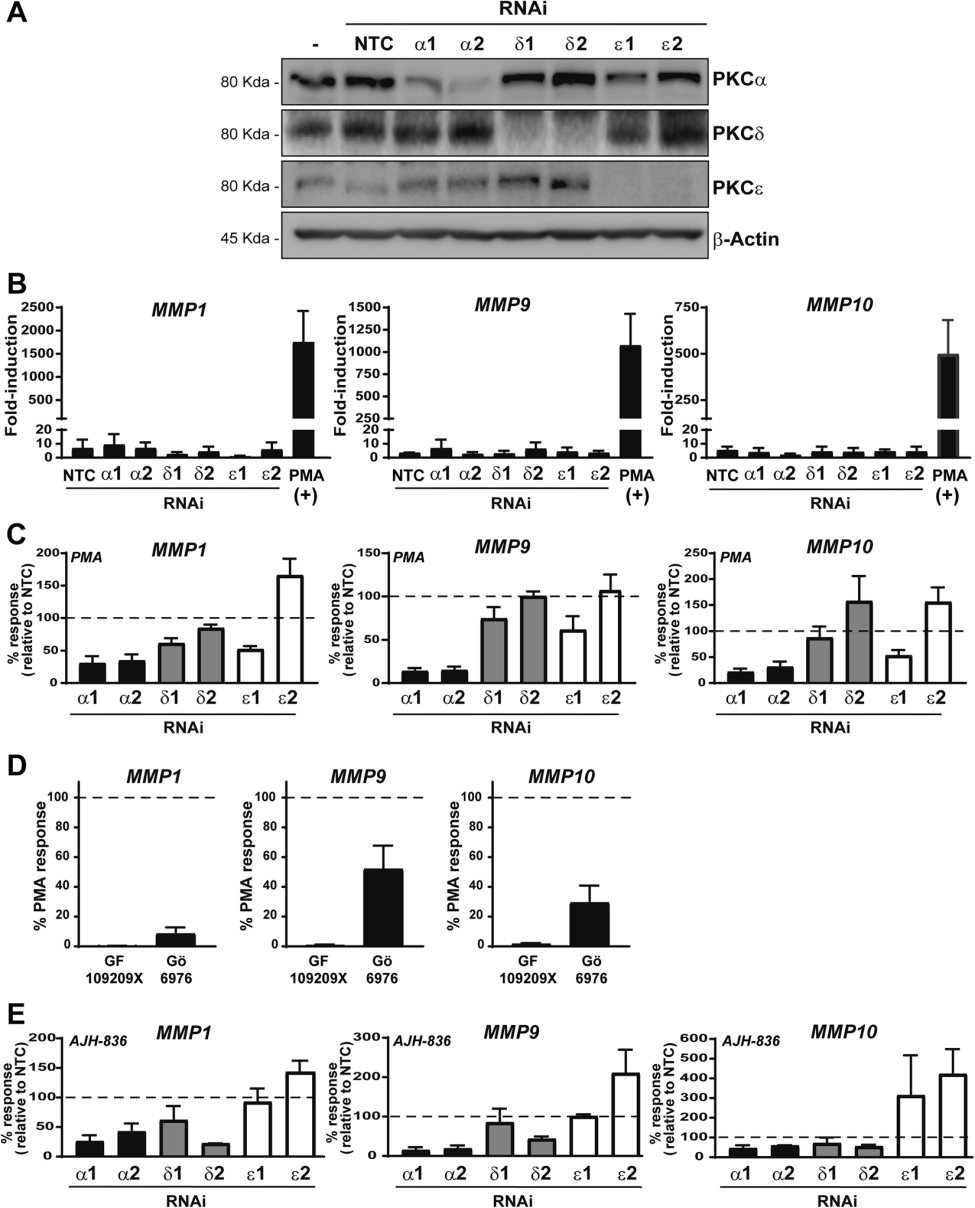
Effect of PKC isozyme RNAi on PMA-induced expression of metalloprotease genes. A549 cells were transfected with RNAi duplexes for PKCα, PKCδ or PKCε. Twenty-four h later cells were serum starved for an extra 24 h period, and then stimulated with PMA (0.1 μM), AJH-836 (1 μM) or vehicle for 1 h. RNA was extracted 3 h later and reversed transcribed to cDNA. (**A**) Representative Western blot for PKC isozymes subjected to RNAi depletion. (**B**) Effect of PKC isozyme RNAi on basal levels of *MMP1*, *MMP9* and *MMP10* as determined by Q-PCR. PMA was included as a positive control for induction. Results are expressed as fold-induction relative to cells transfected with non-target control (*NTC*) RNAi duplex. Data represents the mean ± S.E.M. of 3 independent experiments. (**C**) Effect of PKC isozyme RNAi depletion on the induction of *MMP1*, *MMP9* and *MMP10* by PMA. Results are expressed as percentage of response relative to NTC (*dotted line*). Data represents the mean ± S.E.M. of 3–5 independent experiments. (**D**) Metalloprotease gene induction by PMA was determined in the presence of either 3 μM GF109203X (*GF*) or Gö6976 (*Gö*), added 30 min before and during PMA treatment. Data represents the mean ± S.E.M. of 3 independent experiments. Full-length blots are presented in Supplementary Fig. 4. (**E**) Effect of PKC isozyme RNAi depletion on the induction of *MMP1*, *MMP9* and *MMP10* by AJH-836. Results are expressed as percentage of response relative to NTC (*dotted line*). Data represents the mean ± S.E.M. of 3–5 independent experiments.

We hypothesized that the differential pattern of gene induction by PMA and AJH-836 may be the consequence of their distinctive selectivity for PKC isozymes. The involvement of individual PKCs expressed in gene induction was examined using RNAi. For optimization, we tested several RNAi duplexes and selected the two most specific for each PKC isozyme, which we named α1, α2, δ1, δ2, ε1 and ε2. Western blot in Fig. 5A shows an effective depletion of PKCα, PKCδ, and PKCε by the corresponding RNAi duplexes in A549 cells, which was greater than 90% in all cases. The only caveat observed upon extensive standardization was a consistent partial depletion of PKCα (~50%) with the ε1 duplex.

In the next step, we examined the effect of silencing individual PKCs on the expression of metalloproteases genes. In the first set of experiments, we observed that knocking down PKCα, PKCδ, or PKCε did not have any significant effects on the basal expression levels of *MMP1*, *MMP9* or *MMP10* (Fig. 5B). Silencing PKCα with either α1 or α2 RNAi duplexes markedly reduced PMA induction of *MMP1*, *MMP9*, and *MMP10* by ~70%, ~90%, and ~80%, respectively (Fig. 5C). PKCδ RNAi depletion caused only minor changes in *MMP1* induction by PMA, without any noticeably effects on the up-regulation *MMP9* or *MMP10* expression. With regard to PKCε depletion, we did not find any inhibition in PMA-induced up-regulation of metalloprotease genes by the ε2 RNAi duplex (indeed there is a slight increase for *MMP-1* and *MMP-10*). However, a minor inhibitory effect was observed for the ε1 duplex (Fig. 5C). This is consistent with the partial “off-target” PKCα silencing caused by this PKCε RNAi duplex, as we showed in Fig. 5A. In accordance with the results from the siRNA treatments, pre-treatment with the “pan” PKC inhibitor GF109203X or the cPKC inhibitor Gö6976 inhibited the induction of metalloprotease genes by PMA, again emphasizing the role of PKCα (Fig. 5D). Altogether, we conclude that PKCα is the most prominent PKC involved in the induction of metalloprotease genes in response to the phorbol ester. We also found that the small induction of *MMP1*, *MMP9*, and *MMP10* caused by AJH-836 is reduced in PKCα-depleted A549 cells (Fig. 5E). This may be explained by the marginal activation of PKCα caused by this DAG-lactone at the concentration used (which still fully activates PKCε), as we described in our previous study^36^.

### Analysis of *FST* and *CCL20* induction by PMA and AJH-836 in A549 cells

**Figure 6:**
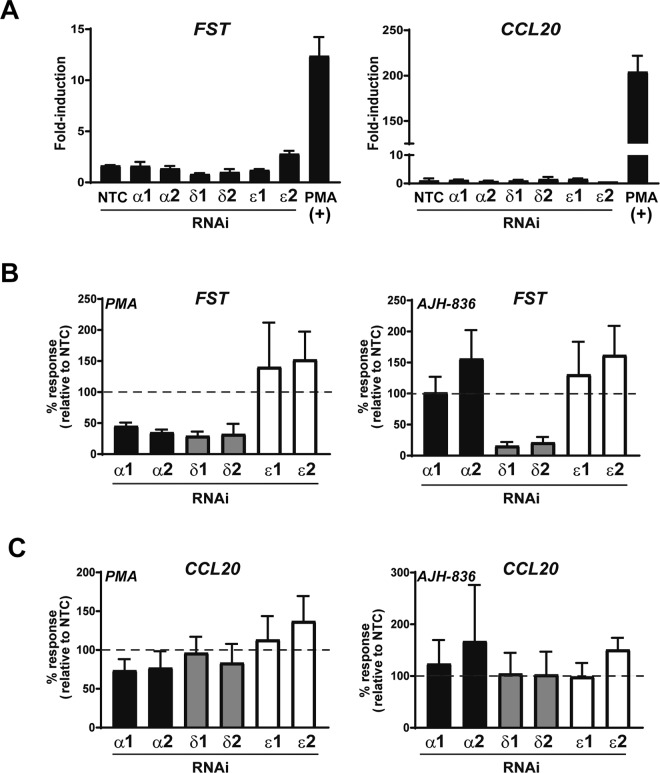
Effect of PKC isozyme RNAi depletion on PMA- and AJH-836-induced expression of *FST* and *CCL20*. A549 cells were transfected with RNAi duplexes for PKCα, PKCδ or PKCε. Twenty-four h later cells were serum starved for an extra 24 h period, and then stimulated with either PMA (0.1 μM) or vehicle for 1 h. RNA was extracted 3 h later and reversed transcribed to cDNA. (**A**) Effect of PKC isozyme RNAi on basal levels of *FST* and *CCL20* as determined by Q-PCR. PMA was included as a positive control for induction. Results are expressed as fold-induction relative to cells transfected with non-target control (*NTC*) RNAi duplex. Data represents the mean ± S.E.M. of 3 independent experiments. (**B**) Effect of PKC isozyme RNAi depletion on induction of *FST* by PMA and AJH-836. Results are expressed as percentage of response relative to NTC (*dotted line*). Data represents the mean ± S.E.M. of 3–5 independent experiments (**C**) Effect of PKC isozyme RNAi depletion on induction of *CCL20* by PMA and AJH-836. Results are expressed as percentage of response relative to NTC (*dotted line*). Data represents the mean ± S.E.M. of 3–5 independent experiments.

As described above, only a small number of genes were induced by AJH-836 relative to PMA. Two of the top genes selected for this analysis (*FST* and *CCL20*) were equally up-regulated by PMA and the nPKC selective analogue AJH-836. To investigate the relative contribution of PKC isozymes to these responses, we examined the effect of knocking down individual PKCs. None of the RNAi duplexes used to silence PKCα, PKCδ or PKCε had any effect on the basal expression of *FST* or *CCL20* (Fig. 6A). *FST* induction by PMA and AJH-836 display a different pattern of sensitivity to silencing of individual PKC isozymes. Specifically, whereas PKCα and PKCδ RNAi inhibited *FST* up-regulation by PMA, only PKCδ RNAi was able to reduce the effect of the AJH-836 (Fig. 6B). Thus, consistent with its selectivity for nPKCs, AJH-836 activates *FST* induction via PKCδ, whereas PMA exerts its effect through the activation of PKCα and PKCδ. There was no effect of PKCε RNAi on the induction of *FST* by either agent.

Analysis of *CCL20* expression appeared to show, unexpectedly, that no changes in *CCL20* up-regulation by either PMA or AJH-836 were observed upon silencing of PKCα, PKCδ, or PKCε, individually (Fig. 6C). Although this could suggest that none of these PKC isoforms were involved in its regulation, the alternative interpretation is that CCL20 induction could be mediated by multiple PKC isoforms and was sufficiently sensitive to induction so that the remaining isoforms that were not knocked down in each instance were able to drive complete induction.

### PKCα mediates PMA-induced production of MMP-9 in A549 cells

**Figure 7:**
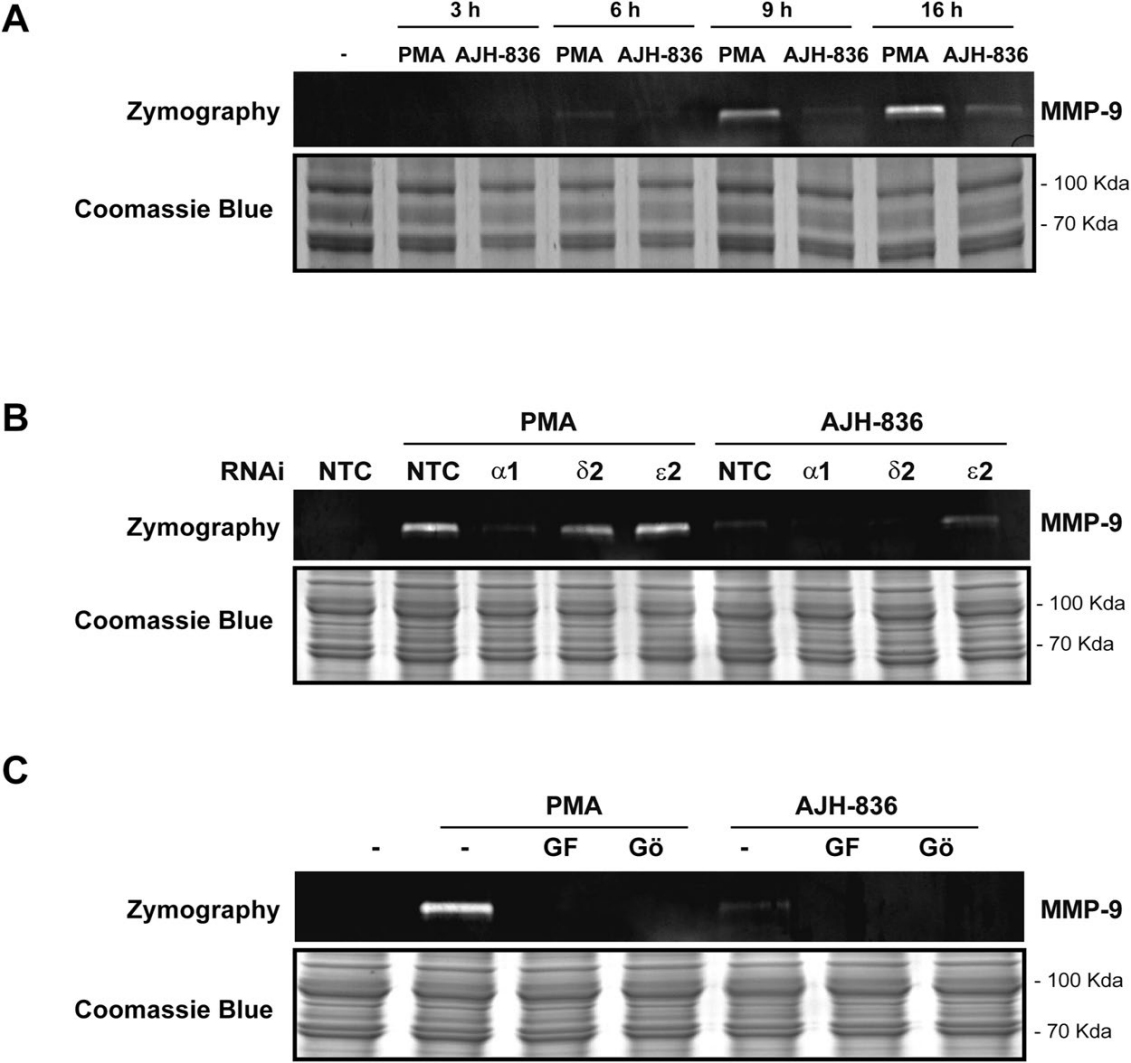
PKCα induces MMP-9 production in A549 cells. (**A**) Cells were treated with PMA (0.1 μM), AJH-836 (1 μM) or vehicle for 1 h and at different times the conditioned medium was collected. MMP-9 activity was determined using zymograms, as described in “Materials and Methods”. As a loading control for the conditioned media, Commassie Blue stained genes are included. (**B**) MMP-9 activity was determined at 16 h after treatment with PMA in A549 cells subjected to PKC isozyme RNAi. Representative experiments are shown. Similar results were observed in 2 additional experiments. (**C**) MMP-9 activity was determined at 16 h after treatment with PMA or AJH-836 in A549 cells, in the presence of either 3 μM GF109203X (*GF*) or Gö6976 (*Gö*), added 30 min before and during PMA treatment. A representative experiment is shown. Similar results were observed in at least two separate experiments. Full-length blots are presented in Supplementary Fig. 5.

In order to address the functional relevance of the induction of metalloprotease genes by PKCα in lung cancer cells, we determined MMP-9 activity by zymography, using conditioned medium collected from A549 cells treated with either PMA or AJH-836. A time-course analysis after PMA treatment revealed that MMP-9 activity could be detected as early as 6 h after PMA treatment, with maximum activity at 16 h (Fig. 7A). Longer times failed to produce a further increase in MMP-9 production (data not shown). On the other hand, and in agreement with gene induction studies, AJH-836 caused only a marginal elevation in MMP-9 activity under the same experimental conditions.

Finally, we determined the effect of silencing individual PKCs on the production of MMP-9. Zymogram analysis of A549 cells subjected to PKCα knock down revealed that in response to PMA the release of MMP-9 to the conditioned medium was essentially blunted (Fig. 7B). Similar results were observed by measuring MMP-9 production in the conditioned medium using an MMP-9 fluorometric assay (Fig. S2). On the other hand, silencing PKCδ or PKCε did not inhibit the production of MMP-9 in response to the phorbol ester (Fig. 7B). In accordance with these results, we found complete inhibition of PMA-induced MMP-9 production in A549 cells by the cPKC inhibitor Gö6976 or the “pan” PKC inhibitor GF109203X (Fig. 7C). Altogether, these results demonstrate a major role for PKCα in MMP-9 production in A549 cells, and highlight the differential involvement of individual PKCs in metalloprotease gene induction.

## Discussion

In the present study, we report the first comparative gene profiling analysis of DAG-lactones and the prototypical PKC activator PMA. We found significant differences in the pattern of gene expression induced by individual compounds, a distinction that emphasizes their unique patterns of target specificity as well as underscores the distinctive involvement of PKC isozymes in the control of gene expression.

Most PKC activators derived from natural sources, such as phorbol esters and bryostatins, display characteristic patterns of biological responses, probably reflecting their distinctive ability to activate and/or relocalize PKC isozymes in a cellular context despite their relative lack of isozyme selectivity *in vitro*^24,41,42^. As evidenced from animal studies, agents such as bryostatin 1 and the deoxyphorbol ester prostratin have anti-tumor effects that stand in marked contrast to the typical tumor promoter activity of phorbol esters^43^. Some of the C1 domain ligands with promising therapeutic potential are either scarce in nature or difficult to synthetize, thus limiting their accessibility for basic and clinical studies. Thus, efforts have been made to generate simpler structures capable of activating PKCs. A major challenge has been the generation of ligands that can specifically discriminate C1 domains, thus conferring selectivity for the activation of individual PKCs. Whereas physiological DAGs do not display selectivity for PKC isozymes^22^, their cyclization to rigid lactone structures represents a promising strategy towards the generation of C1 domain ligands with potential selectivity. Indeed, we recently identified the DAG-lactone AJH-836 as a ligand with preferential selectivity towards nPKCs *in vitro* and in cellular models relative to cPKCs^36^.

The strikingly different patterns of gene expression induced by AJH-836 and PMA treatment uncovered unique arrays of transcriptional networks regulated by discrete PKC isozymes. Notably, AJH-836 regulated only a small subset of PMA-regulated genes in A549 lung cancer cells. This suggests a limited involvement of nPKCs in the control of gene expression in this model, but rather a major contribution of PKCα. A representative example of an nPKC-regulated gene outlined in our study is *FST*, which is induced by AJH-836 via PKCδ. PMA on the other hand modulates the expression of a large number of genes. PKCα has been previously implicated in gene induction and repression, involving a range of transcription factors such as NF-κB and Elk1, and transcriptional repressors such as Id1^44–46^. Notably, our bioinformatics analysis revealed significant involvement of NF-κB as well as enrichment of other pathways strongly linked to NF-κB, including cytokine-related pathways. Moreover, we found a significant enrichment of NF-κB binding sites among PMA-regulated genes. The involvement of PKCα as a central signaling node in cancer cells has been postulated by Weinberg and co-workers, who established this cPKC as a regulator of specific members of the Fos family of transcription factors to control key steps in the invasion process^47^. Our analysis also identified AP1 as a key transcription factor regulated by PMA, consistent with early studies involving Fos and Jun dimers in phorbol ester-induced control of gene expression^48^. Consistent with the involvement of PKCα in metastatic dissemination of cancer cells^49–52^, we observed that this cPKC mediates PMA induction of metalloproteases widely implicated in the degradation of extracellular matrix components, whereas nPKCs δ and ε are dispensable. As we showed in our study, *MMP1*, *MMP9* and *MMP10* genes are elevated in human lung adenocarcinomas relative to normal tissue.

Based on the differential regulation of gene expression observed between PMA and AJH-836 in A549 cells, we conclude that PKCα is the major PKC driving gene expression in this cellular model. This was somehow unexpected based on our previous genome wide-analysis in prostate cancer cell models, in which PKCδ and to a lesser extent PKCε represent the main PKC drivers of gene expression^37^. The relative contribution of individual PKCs to transcriptional regulation may be dictated by their relative expression in a given cell type and/or their specific coupling to signaling pathways, including MEK/Erk, NF-κB, and Stat3, which are known to control the induction and repression of different subsets of genes^11,38,53–55^.

An interesting observation is that *CCL20*, which is induced by both PMA and AJH-836, is insensitive to the silencing of individual PKCs. One model is that several PKC isoforms could have redundant activity for induction of this gene and that the activation signal is saturated under our assay conditions, so that whatever PKC isoform that was not knocked down was sufficient for a full response. An alternative attractive hypothesis is that *CCL20* exemplifies a subset of phorbol ester/DAG-regulated genes modulated in a PKC-independent manner. It is well established that C1 domains are also present in other kinases, such as PKDs, as well as in proteins unrelated to kinases, such as RasGRP (Ras exchange factors) and chimaerin (Rac GTPase activating factors)^1,3^. There is evidence that pathways regulated by “non-PKC” phorbol ester/DAG receptors control gene expression^56–58^, however the actual transcriptional networks regulated by these proteins have not been yet characterized. Testing this hypothesis would require targeted silencing of individual “non-PKC” phorbol ester/DAG receptors, a demanding task due to the large size of this family, which comprises at least 22 members^3^. Of note, recent studies identified DAG-lactones with selectivity towards RasGRPs^59,60^, which would be highly useful in this setting. This emphasizes the rich opportunities afforded by synthetic design of new DAG-lactone analogues.

Another interesting feature of our study was the unforeseen pattern of gene expression observed upon treatment with AJH-1512. The original goal in our study was using this DAG-lactone as an AJH-836 control, for two reasons. First, AJH-1512 has equal affinity for cPKCs and nPKCs both *in vitro* and in cellular models, and second, at the concentration used, it causes minimal PKC activation in cells^36^. Regardless, AJH-1512 caused a surprisingly strong effect on gene expression. We speculate that, as indicated above, genes commonly activated by PMA and AJH-1512 may represent a subset of genes regulated by “non-PKC” phorbol ester receptors. It has been noticed in a very recent study that some DAG-lactones can bind to RasGRPs at much lower concentrations than PKCα and even PKCε^61^. Another unexpected observation was that a significant number of genes regulated by AJH-1512 were not modulated by PMA. A likely explanation is that AJH-1512 may be also activating other yet unidentified pathways at concentration in which DAG/phorbol ester receptors are barely activated. This conclusion is supported by strikingly different functional pathway enrichment between PMA and AJH-1512. Clearly, substitutions in the DAG-lactone backbone have great influence on biological responses. Extra caution should be taken in the interpretation of the cellular effects of these compounds until a thorough characterization of targets and pathways is pursued.

In summary, by taking advantage of chemically modified DAG-lactones we were able to elucidate specific roles of individual members of the PKC family on gene expression. Our results not only highlight the relevance of PKCα in the control of the transcriptional regulation of genes relative to the nPKCs in lung cancer cells, but also unveil the intricacies in the biological patterns controlled by the DAG pathway. Due to the widespread implications of phorbol ester/DAG receptors in cancer, neurological and cardiovascular disorders^3,62–64^, the generation of C1 domain ligands with distinctive specificity may represent promising therapeutic leads and pharmacological tools for dissecting the pathophysiological basis of disease.

## Materials and Methods

### Cell lines and materials

Authenticated human A549 lung adenocarcinoma cells were obtained from ATCC (Manassas, VA) and cultured in RPMI medium supplemented with 10% FBS, 2 mM glutamine, 100 U/ml penicillin, and 100 µg/ml streptomycin). PMA was purchased from Sigma-Aldrich (St. Louis, MO) PKC inhibitors GF109203X and Gö6976 were purchased from TOCRIS (Bristol, UK).

### Synthesis of AJH-836 and AJH-1512

The synthesis of AJH-836 and AJH-1512 is reported in detail elsewhere^35^. These agents are described as compounds 104 and 98, respectively, in that study.

### Treatment with PMA and DAG-lactone AJH-836

A549 cells were serum-starved for 18 h and then treated for 1 h with PMA (0.1 μM), AJH-836 (1 μM), AJH-1512 (1 μM), or vehicle (DMSO) in serum-free medium. After washing twice, cells were incubated for 3 h in serum-free medium and processed for analysis of gene expression (RNASeq or Q-PCR).

### RNA interference (RNAi)

For silencing individual PKCs, we used previously validated ON-TARGET Plus RNAi sequences from Dharmacon (Lafayette, CO). Catalog numbers are as follows: J-003523-17-0002 (PKCα), J-003524-08-0002 (PKCδ), and J-004653-08-0002 (PKCε). As a non-target control (NTC), we used D-001810-02-05 ON-TARGETplus non-targeting siRNA #2,5. RNAi duplexes were transfected using Lipofectamine RNAi Max (Invitrogen, Carlsbad, CA). Experiments were carried out 48 h after transfection.

### RNA isolation and Real Time Quantitative PCR

Total RNA was extracted from subconfluent plates using the RNeasy kit (Qiagen, Valencia, CA) as previously described^65^. Briefly, one μg of RNA per sample was reverse transcribed using the Taqman reverse transcription reagent kit and random hexamers as primers (Applied Biosystems, Branchburg, NJ). Primers for individual genes (MMP-1, MMP-9, MMP-10, CCL20, FST, UBC) were purchased from Applied Biosystems. PCR amplifications were performed using an ABI PRISM 7300 Detection System in a total volume of 20 μl containing Taqman Universal PCR Master Mix (Applied Biosystems), commercial target primers (300 nM), the fluorescent probe (200 nM), and 1 μl of cDNA. PCR product formation was continuously monitored using the Sequence Detection System software version 1.7 (Applied Biosystems). The FAM signal was normalized to endogenous UBC (a housekeeping gene).

### RNA-Seq data analysis

RNA was isolated from subconfluent plates using the RNeasy kit (Qiagen, Valencia, CA) as previously described^65^. RNA concentration and integrity were measured on an Agilent 2100 Bioanalyzer (Agilent Technologies). Only RNA samples with RNA integrity values (RIN) over 8.0 were considered for subsequent analysis. RNA samples were processed for directional RNA-Seq library construction and sequencing at the NGS Core facility of the Perelman School of Medicine, University of Pennsylvania. We performed 100 nt singled-end sequencing using an Illumina HiSeq. 4000 platform and obtained ~20 million reads per sample. The short-sequenced reads were mapped to the human reference genome (hg19) with the RNA-Seq unified mapper (RUM) V2.0.5. 06 (http://cbil.upenn.edu/RUM/). We employed R/Bioconductor packages to accurately calculate the gene expression abundance using the aligned BAM files. Raw data have been submitted to the NCBI GEO database. Briefly, to identify differentially expressed genes (log2 fold change [log2 FC] > ±1, false discovery rate [FDR] <0.05) between treatments and vehicle samples we employed the edgeR Bioconductor package based on the normalized log2 based count per million values.

For functional enrichment analyses, we used ClueGo Cytoscape’s plug-in (http://www.cytoscape.org/) and the InnateDB resource (http://www.innatedb.com/) based on the list of dysregulated transcripts. Over-represented transcription factor binding sites (TFBS) among dysregulated genes were detected using three different resources: Enrichr (ENCODE and ChEA TFBS databases), InnateDB, and oPOSSUM 3.0 (TFBS CisRED database). RNA-Seq expression profiles of 584 normal and lung adenocarcinomas from the TCGA-LUAD project were download from UCSD-Xena resource (https://xena.ucsc.edu/) for further analysis of PMA modulated genes. Data integration and visualization of differentially expressed transcripts were done with R/Bioconductor and the MultiExperiment Viewer software (MeV v4.9).

### Western blots

Western blots were done essentially as previously described^66^. Briefly, A549 cells were harvested in lysis buffer containing 50 mM Tris-HCl, pH 6.8, 10% glycerol, and 2% β-mercaptoethanol. Cell lysates were subjected to SDS-polyacrylamide gel electrophoresis and transferred to polyvinylidene difluoride membranes (Millipore Corporation, Billerica, MA). After blocking with 5% milk or 5% BSA in Tris-buffered saline/0.1% Tween for 1 h, membranes were incubated overnight with the following primary antibodies: anti-PKCα, anti-PKCδ, anti-PKCε (all from Cell Signaling, 1:1,000 dilution, Catalog #2056, #2058, and #2083, respectively), vinculin (Sigma-Aldrich, 1:5,000, catalog #V9131) or β-actin (Sigma-Aldrich, 1:50,000 dilution, catalog #A5441). Membranes were then incubated for 1 h with either anti-mouse (1:1,000 dilution) or anti-rabbit (1:3,000 dilution) secondary antibodies conjugated to horseradish peroxidase (Bio-Rad Laboratories, Hercules, CA). Bands were visualized and subjected to densitometric analysis using an Odyssey Fc system (LI-COR Biotechnology, Lincoln, NE).

### Determination of MMP-9 protein levels in conditioned medium

Conditioned medium from A549 cells subjected to the different treatments was collected at different times (0–24 h), and MMP-9 levels were determined using Novex^TM^ 10% zymogram Plus (gelatin) gels (Invitrogen-Life Technologies). Gels were run in a Tris-glycine SDS running buffer (Invitrogen-Life Technologies). After electrophoresis, gels were renatured, stained, and developed using the conditions described by the manufacturer. MMP-9 levels were also determined using the SensoLyte ® Plus 520 MMP-9 Assay kit (AnaSpec, Inc. Fremont, CA).

### Statistical analysis

Analysis of variance was performed using GraphPad Prism software built-in analysis tools. The confidence interval was set to 95%. A p < 0.05 was considered statistically significant.

## Supplementary information

Supplemental Information

Supplemental Table 1

Supplemental Table 2
