# Supplementary material for: Differential Regulation of Gene Expression in Lung Cancer Cells by Diacyglycerol-Lactones and a Phorbol Ester Via Selective Activation of Protein Kinase C Isozymes
Source: Sci Rep. 2019 Apr 15;9:6041. doi: 10.1038/s41598-019-42581-4 (PMC6465381; doi:10.1038/s41598-019-42581-4)
Supplement: Supplementary file 1 — Supplemental Information [file 41598_2019_42581_MOESM1_ESM.pdf]

DIFFERENTIAL REGULATION OF GENE EXPRESSION IN LUNG CANCER CELLS BY DIACYGLYCEROL-LACTONES AND A PHORBOL ESTER VIA SELECTIVE ACTIVATION OF PROTEIN KINASE C ISOZYMES

Mariana Cooke, Victoria Casado-Medrano, Jihyae Ann, Jeewoo Lee, Peter M. Blumberg, Martin C. Abba and Marcelo G. Kazanietz

SUPPLEMENTAL MATERIAL

Figure S1. Functional analysis of AJH-1512-regulated genes. Network of functionally enriched pathways and genes differentially expressed by AJH-1512 treatment relative to vehicle. *Red*, up-regulated genes.

Figure S2. Involvement of PKCα in MMP-9 production. Cells subjected to PKCα RNAi were treated with either PMA (0.1 μM) or AJH-836 (1 μM) for 1 h, and conditioned medium was collected at 16 h. MMP-9 activity was determined using a fluorometric assay as described in "Materials and Methods". Results are expressed as mean ± S.E.M. (n=3). *NTC*, non-target control. *RFU*, relative fluorescence units.

Figure S3. Effect of PKC isozyme RNAi on PMA-induced expression of metalloprotease genes. Full-length images for blots shown in Figure 5A.

Figure S4. PKCα induces MMP-9 production in A549 cells. Full-length images for gels shown in Figure 7.

Suppl. Table 1. Expression profiles for genes induced by PMA, AJH-836, and AJH-1512. The mRNA fold-induction values for all genes induced by the different treatments are presented, grouped into either up- or down-regulated genes.

Suppl. Table 2. Genes up-regulated and down-regulated both by PMA in A549 cells and in human lung adenocarcinomas. The mRNA fold-induction values for all genes in tumors are listed.

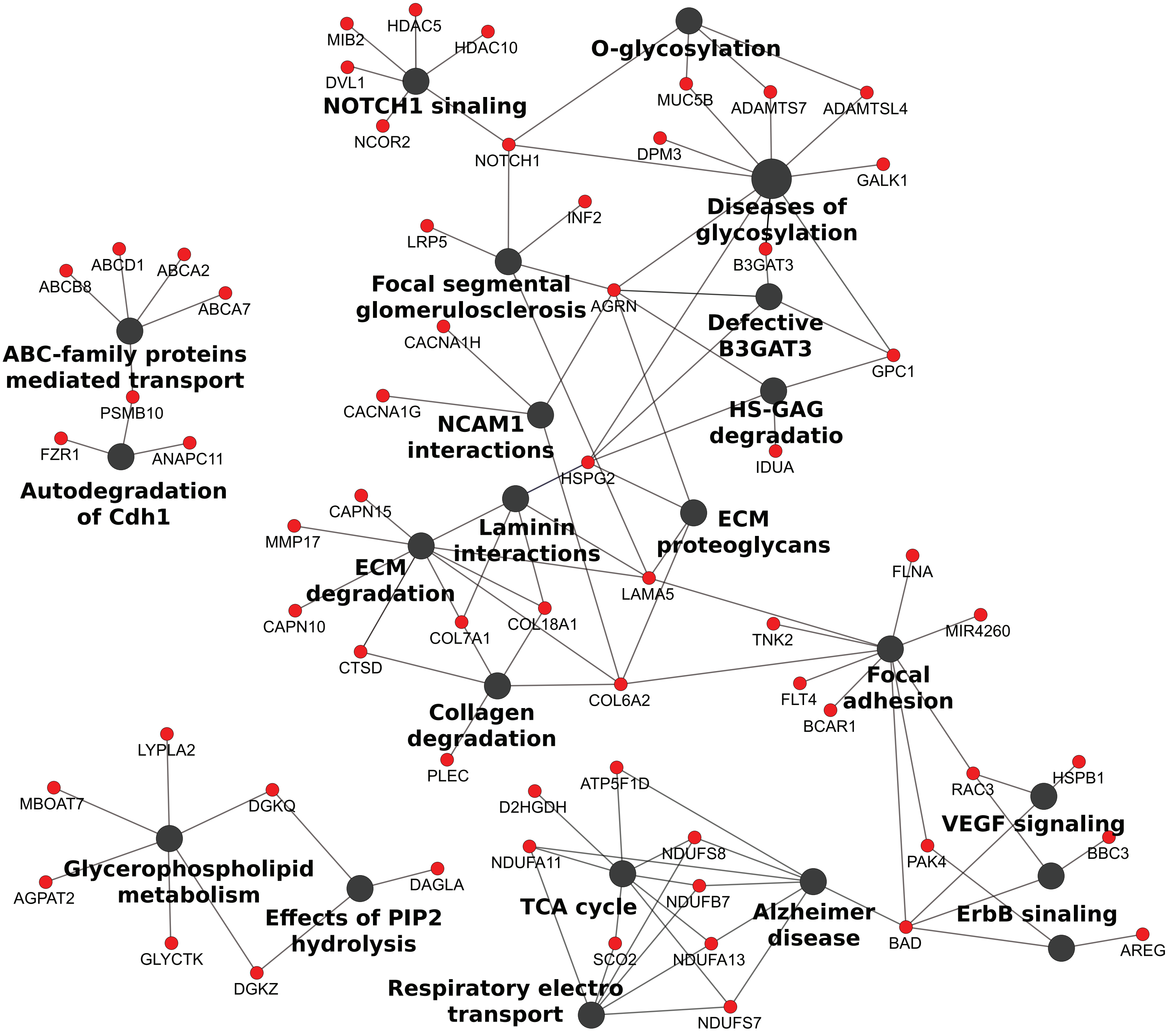

Figure S1. Functional analysis of AJH-1512-regulated genes. Network of functionally enriched pathways and genes differentially expressed by AJH-1512 treatment relative to vehicle. *Red*, up-regulated genes.

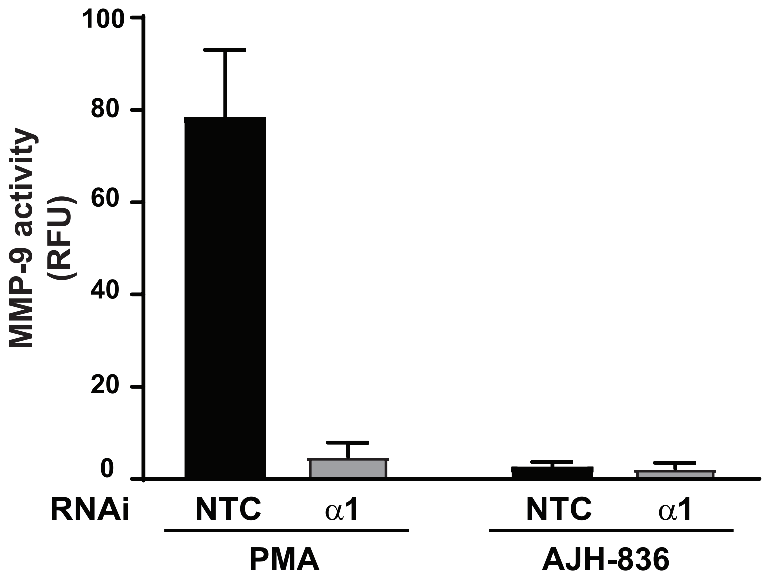

Figure S2. Involvement of PKCα in MMP-9 production. Cells subjected to PKCα RNAi were treated with either PMA (0.1 μM) or AJH-836 (1 μM) for 1 h, and conditioned medium was collected at 16 h. MMP-9 activity was determined using a fluorometric assay as described in "Materials and Methods". Results are expressed as mean ± S.E.M. (n=3). *NTC*, non-target control. *RFU*, relative fluorescence units.

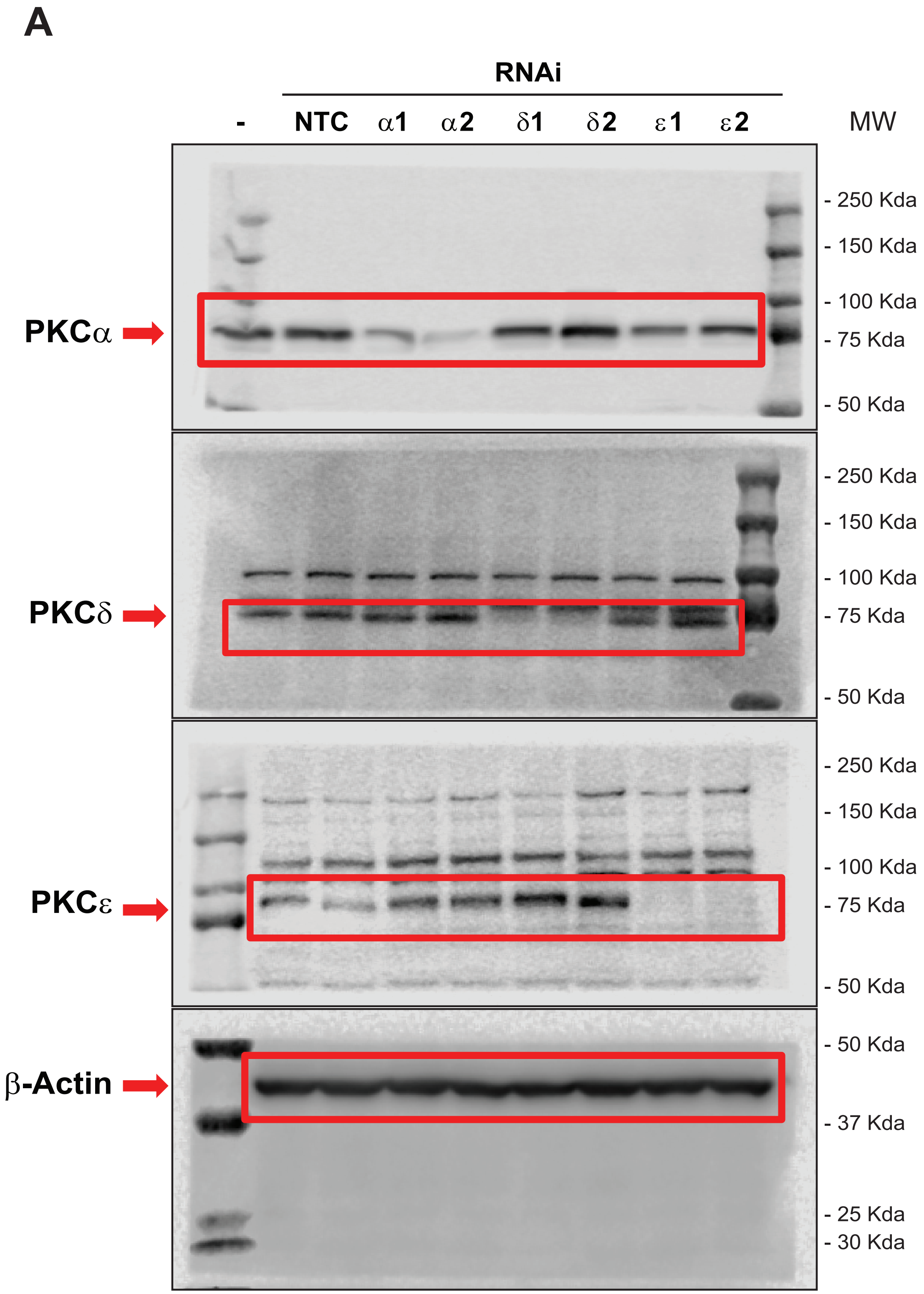

Figure S3. Effect of PKC isozyme RNAi on PMA-induced expression of metalloprotease genes. Full-length images for blots shown in Figure 5A.

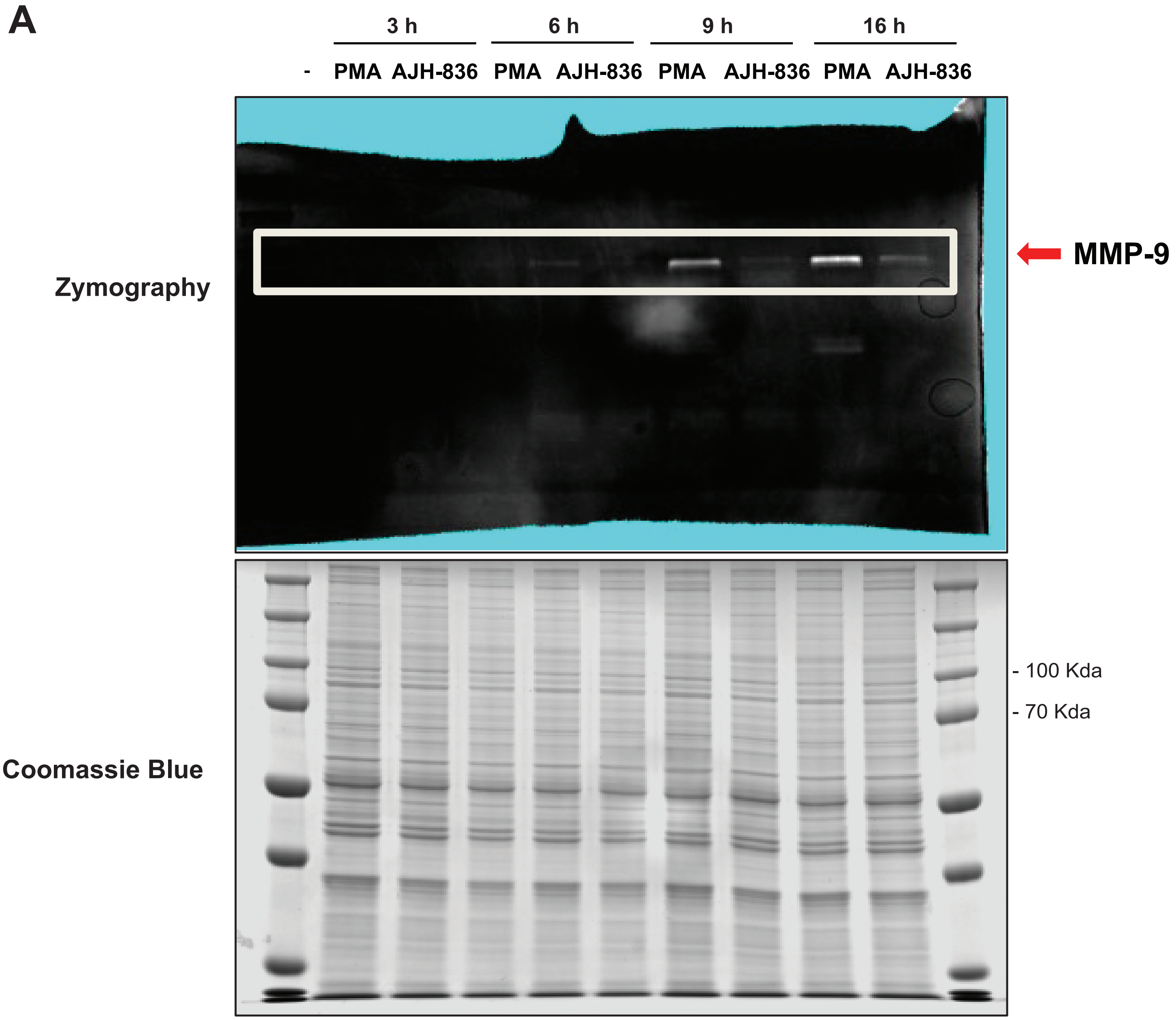
A
3 h
6 h
9 h
16 h
-
PMA
AJH-836
PMA
AJH-836
PMA
AJH-836
PMA
AJH-836
Zymography
MMP-9
Coomassie Blue
- 100 Kda
- 70 Kda

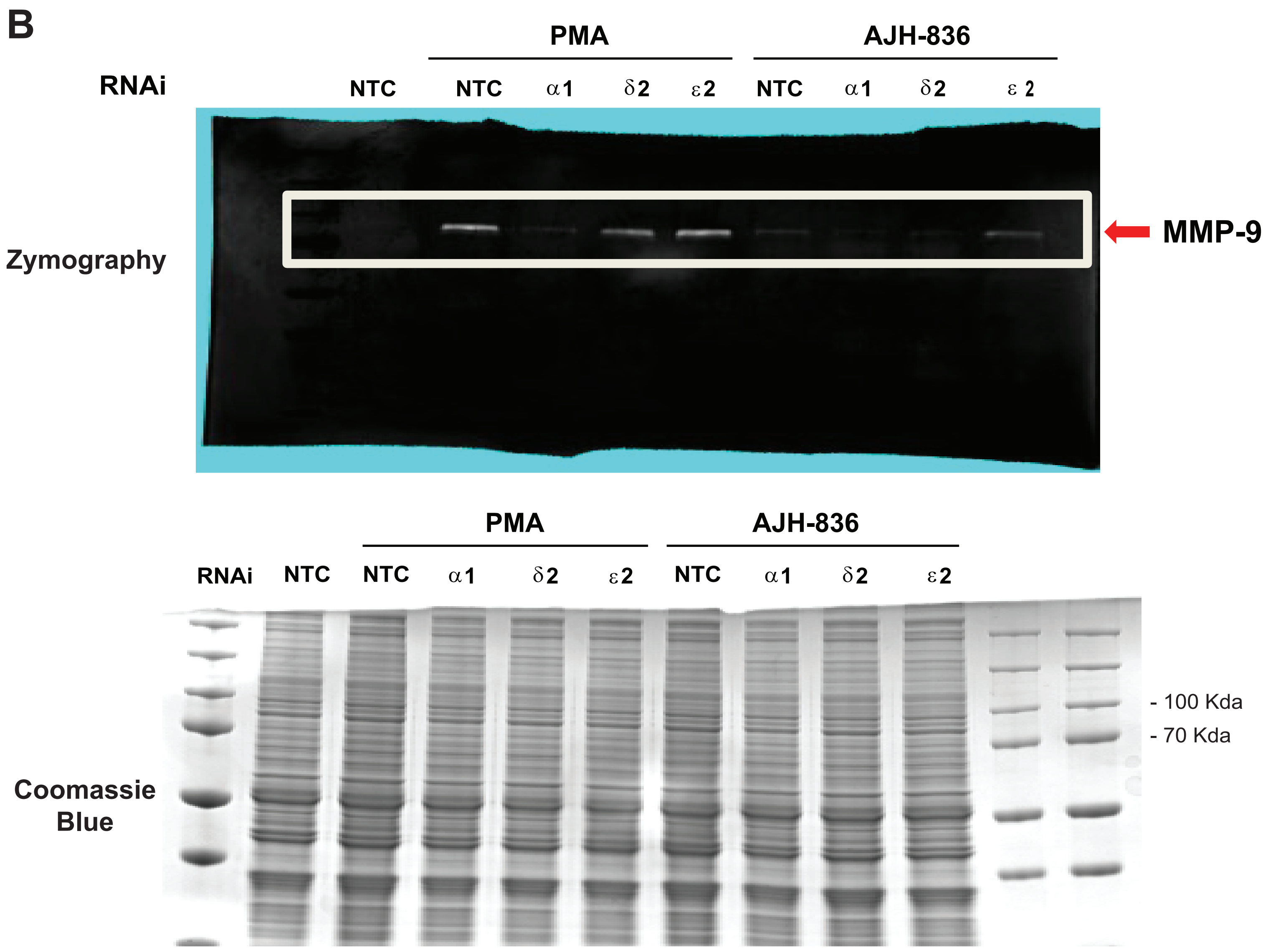
B
PMA
AJH-836
RNAi
NTC
NTC
α1
δ2
ε2
NTC
α1
δ2
ε 2
Zymography
MMP-9
PMA
AJH-836
RNAi
NTC
NTC
α1
δ2
ε2
NTC
α1
δ2
ε2
Coomassie Blue
- 100 Kda
- 70 Kda

C

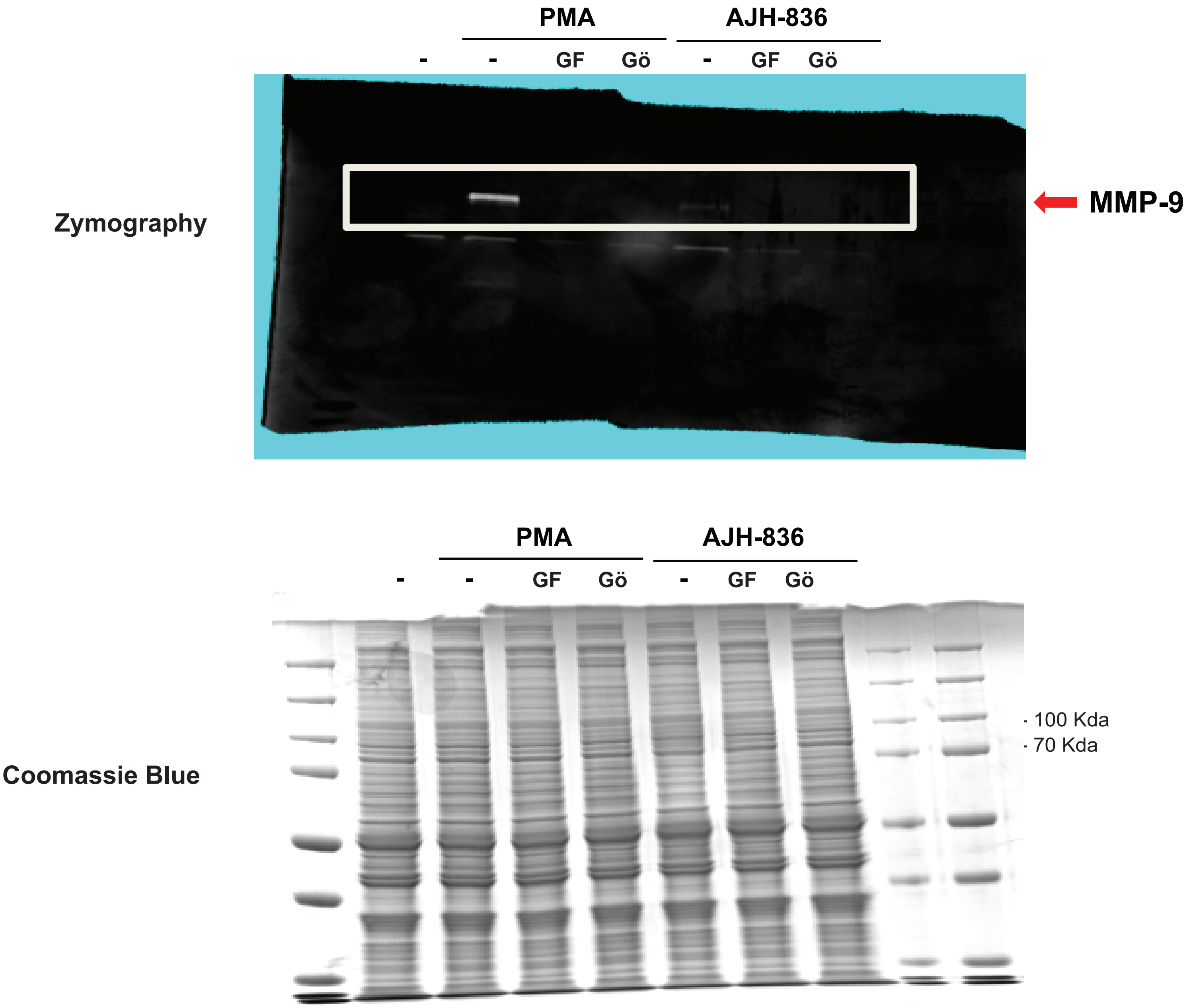

Figure S4. PKCα induces MMP-9 production in A549 cells. Full-length images for gels shown in Figure 7.
